# Molecular Characterization of KPC-2 and CMY AmpC Beta Lactamases in Ceftazidime-Avibactam Resistant Klebsiella pneumoniae in a Tertiary Care Hospital

**DOI:** 10.7759/cureus.95576

**Published:** 2025-10-28

**Authors:** Usama Haseeb, Samana Mukhtar, Faryal Yunus, Masham Mukhtar, Masood Rabbani, Haroon Akbar, Aamir Ghafoor

**Affiliations:** 1 Institute of Microbiology, University of Veterinary and Animal Sciences, Lahore, PAK; 2 Department of Pathology, National Hospital and Medical Centre, Lahore, PAK; 3 Department of Internal Medicine, Allama Iqbal Medical College, Jinnah Hospital Lahore, Lahore, PAK; 4 Department of Microbiology, National Hospital and Medical Centre, Lahore, PAK; 5 Department of Parasitology, University of Veterinary and Animal Sciences, Lahore, PAK

**Keywords:** ceftazidime-avibactam, cmy ampc beta-lactamase, klebsiella pneumoniae, kpc, multi-drug resistance

## Abstract

Introduction

Multidrug-resistant (MDR) *Klebsiella pneumoniae* poses significant treatment challenges, particularly in intensive care settings. Ceftazidime-avibactam (CZA) has emerged as a key option against extended-spectrum beta-lactamase (ESBL) and *K. pneumoniae *carbapenemase (KPC) producing strains; however, increasing resistance, frequently attributed to CMY AmpC β-lactamases and porin loss, threatens its efficacy. Despite clinical use in Pakistan, local molecular data on resistance mechanisms remain limited. This study assessed antimicrobial resistance patterns, determined CZA minimum inhibitory concentrations (MICs), and screened for *bla*_KPC-2_ and *bla*_CMY_ genes in resistant *K. pneumoniae* isolates from a tertiary care center.

Materials & methods

This cross-sectional study included 200 non-duplicate clinical specimens collected from various departments of the National Hospital and Medical Centre, Lahore. From these, 120 *K. pneumoniae* isolates were recovered, which were subsequently identified and analyzed using standard microbiological methods and confirmed 2023 guidelines of the Clinical and Laboratory Standards Institute (CLSI) with the VITEK 2 Compact system (bioMérieux SA, Marcy-l'Étoile, France). Antimicrobial susceptibility testing, including CZA MIC determination, was conducted. Polymerase chain reaction was performed on CZA non-susceptible isolates for the detection of *bla_KPC-2_* and *bla_CMY_* genes, with sequencing confirmation of positive amplicons. Data on specimen distribution and MIC profiles were analyzed using IBM SPSS Statistics for Windows, version 20 (IBM Corp., Armonk, New York, United States), GraphPad Prism version 9 (Dotmatics, Boston, Massachusetts, United States), and Microsoft Excel (Microsoft Corporation, Redmond, Washington, United States).

Results

From 200 clinical specimens, 120 non-duplicate *K. pneumoniae* isolates were recovered, mainly from urine (26.7%), blood (21.7%), and respiratory samples. Samples were from intensive care unit (ICU) patients (41.7%), followed by the high-dependency unit (HDU) (28.3%) and critical care unit (CCU) (11.7%). Antimicrobial susceptibility testing revealed extensive multidrug resistance, with resistance rates ranging from 93.3% to 99.2% against β-lactams, cephalosporins, carbapenems, and fluoroquinolones. CZA resistance was identified in 30.8% (n = 37) of isolates, while tigecycline (9.2%) and fosfomycin (7.5%) showed the highest susceptibility.

CZA MIC analysis classified 63.3% as susceptible and 30.8% as resistant, with resistant isolates exhibiting a significantly elevated mean MIC (91.46 ± 24.36 µg/mL; p < 0.05). Among the 44 CZA non-susceptible isolates, *bla_CMY_* was detected in 36.4% (n = 16), whereas *bla_KPC-2_* was not detected in any case. Isolates positive for *bla*_CMY_* *AmpCF were resistant to CZA, with a statistically significant association (p < 0.05) between the presence of the gene and resistance. Sanger sequencing confirmed 99.3% similarity with *bla*_CMY-2_.

Conclusion

CMY AmpC β-lactamase emerged as the predominant mechanism of CZA resistance in *K. pneumoniae,* highlighting the need for routine AmpC screening, prudent use of alternatives such as cefiderocol, tigecycline, or fosfomycin, and sustained genomic surveillance to guide therapy and infection control.

## Introduction

The rapid emergence and global dissemination of antimicrobial resistance (AMR) have become a critical concern in modern medicine, posing a significant threat to the effectiveness of existing therapeutic regimens and representing one of the most urgent challenges in clinical microbiology and infectious disease management in the 21st century [1].

*Klebsiella pneumoniae*, a gram-negative rod (GNR) and clinically significant member of the ESKAPE (*Enterococcus faecium, Staphylococcus aureus, Klebsiella pneumoniae, Acinetobacter baumannii, Pseudomonas aeruginosa*, and *Enterobacter species*) group of multidrug-resistant (MDR) organisms, has emerged as a major nosocomial pathogen. It is frequently associated with high-burden healthcare-associated infections, including bacteremia, lower respiratory tract infections, and urinary tract infections, particularly among critically ill and immunocompromised patients. Its increasing prevalence and resistance to multiple antimicrobial agents underscore its role in the global threat of hospital-acquired infections [2].

AmpC is an enzyme a permanently β-lactamase, synthesized by certain bacterial species, and belongs to Class C and Group 1. A cross-sectional study conducted for three years at the Children’s Hospital demonstrated that the high frequency of Enterobacteriaceae strains was associated with a higher frequency of the plasmid-mediated β-lactamase gene, *bla_CMY-2_*. It appears that the detection of AmpC β-lactamases is a critical process in terms of guiding appropriate antimicrobial therapy [3].

As a last resort, carbapenems are used to treat severe infections caused by MDR bacteria. Carbapenemases are enzymes that belong to the Ambler Classes A (serine-based enzymes), B (metallo-beta-lactamases), and D (OXA-type enzymes) beta-lactamases that block beta-lactam antibiotics, including carbapenems. They swiftly bind to the penicillin-binding proteins of gram-negative bacteria after penetrating their cell wall via outer membrane proteins, or porins. *K. pneumoniae* carbapenemase (KPC) is a plasmid-encoded Ambler Class A enzyme. Their existence in *K. pneumoniae* was originally documented in the United States in 1996. Within a few years, KPC producers have spread worldwide and are now present in a wide range of GNRs. More recently, it has been shown that KPC and other carbapenemases coexist in *K. pneumoniae* [4].

CZA represents a significant advancement in antimicrobial chemotherapy, combining ceftazidime, a third-generation cephalosporin, with avibactam, a non-β-lactam diazabicyclooctane (DBO) β-lactamase inhibitor. This synergistic combination exhibits potent activity against a broad range of β-lactamase-producing Enterobacterales, including class A serine carbapenemases (e.g., KPC), class C cephalosporinases (AmpC), and select class D carbapenem-hydrolyzing enzymes such as the OXA-48-like group [5,6]. Since its approval by the United States Food and Drug Administration (FDA) in 2015, CZA has become a cornerstone in the treatment of infections caused by MDR bacteria, particularly those resistant to carbapenems and extended-spectrum β-lactams. Its clinical utility in managing both nosocomial and community-onset infections has positioned it as a critical option in the era of escalating antimicrobial resistance [7].

Furthermore, overexpression of resistance-nodulation-division (RND) family efflux pumps (e.g., AcrAB-TolC) has been found to expel both ceftazidime and avibactam, significantly reducing intracellular drug concentrations and further compromising antimicrobial efficacy [8].

Resistance to CZA has rapidly emerged in *K. pneumoniae*, particularly among KPC producing strains. In clinical isolates from China and the United States, CZA resistance has been frequently linked to mutations in the Ω-loop region of KPC enzymes, including variants such as KPC‑33, KPC‑44, KPC‑86, and KPC‑129, which reduce avibactam affinity and restore ceftazidime hydrolysis. Concurrent outer membrane permeability defects, primarily truncations or insertions in the major porins ompK35 and ompK36, such as the Gly-Asp insertion in OmpK36 loop L3, operate synergistically with omega-loop mutations to elevate minimum inhibitory concentrations (MICs) and promote high-level CZA resistance [9].

In South Asia and the Middle East, CZA resistance is increasingly driven by plasmid-borne CMY AmpC β-lactamases (e.g., CMY-2, CMY-178), often independent of Ω-loop mutations or porin loss. This underscores a regional divergence in resistance mechanisms from the KPC-dominated profiles seen in Western settings [10].

In Pakistan, molecular surveillance of CZA resistance remains limited. While *bla_NDM_* and *bla_OXA-48_* are frequently identified and *bla_KPC_* sporadically reported, plasmid-mediated AmpC β-lactamases, particularly *bla_CMY_*, remain largely uncharacterized in clinical *K. pneumoniae*. A recent pediatric study documented approximately 70% CZA resistance among carbapenem-resistant Enterobacterales; however, the molecular basis of resistance was not elucidated, impeding antimicrobial stewardship and infection control efforts [11].

Emerging regional data highlight a shift in the molecular epidemiology of CZA resistance. Unlike Western settings, where resistance is predominantly KPC-driven, isolates from South Asia and the Middle East increasingly harbor CMY AmpC enzymes (e.g., CMY-2, CMY-178) capable of mediating resistance independently of porin loss or KPC mutations. This evolving resistance mechanism underscores the necessity of incorporating AmpC-focused molecular diagnostics into routine surveillance and guiding region-specific therapeutic strategies.

## Materials and methods

Study design and ethical approval

This study was conducted at the Institute of Microbiology, University of Veterinary and Animal Sciences (UVAS), Lahore, Pakistan, from September 2024 to May 2025, following formal approval from the Institutional Review Committee for Biomedical Research, UVAS (approval number: 344/IRC/BMR). Permission for the collection of clinical specimens was granted by the Microbiology Laboratory Pathology Department, National Hospital & Medical Centre, Lahore, Pakistan (Ref. No. 189/LAB/NHMC).

Sample size

The sample size was calculated using the standard formula for estimating a population proportion in a cross-sectional study:

$n = \frac{Z^2 P q}{d^2}$, where Z represents the Z-score corresponding to a 95% confidence interval (1.96), P denotes the estimated prevalence of CZA resistance in *K. pneumoniae* (assumed to be 20% based on prior literature [12]), and d is the desired precision (0.08).

Based on these parameters, the minimum required sample size was calculated to be 96 isolates. In this study, 120 non-duplicate *K. pneumoniae* isolates were recovered from 200 clinical specimens, thereby fulfilling the sample size requirement and providing sufficient statistical power for subsequent analyses.

Sample collection

A total of 200 clinical specimens including blood, urine, sputum, bronchoalveolar lavage, tracheal secretions, pus, and wound swabs were collected from patients with clinical suspicion of bacterial infections presenting to the Microbiology Department of the National Hospital and Medical Centre, Lahore, Pakistan, and after initial screening by culture and identification 120 *K. pneumoniae* were recovered and included in this study.

Isolation and identification of *K. pneumoniae*

Specimens were processed based on their nature and inoculated onto appropriate culture media, including blood agar, MacConkey agar, chocolate agar, and cysteine-lactose-electrolyte-deficient (CLED) agar, using 90 mm sterile disposable plastic Petri dishes. Standard microbiological protocols were followed for inoculation and incubation under aerobic conditions at 37°C for 24 hours. Post-incubation, bacterial growth was evaluated based on colony morphology and Gram staining characteristics. Preliminary identification of isolates was performed using conventional biochemical tests, including triple sugar iron (TSI) agar, urease, Simmons’ citrate, and sulfide-indole-motility (SIM) tests. All isolates presumptively identified as *K. pneumoniae* were further confirmed using the VITEK® 2 Compact System with GN ID cards (bioMérieux SA, Marcy-l'Étoile, France), which employ a panel of biochemical assays for precise species-level identification and a confidence interval (CI) ≥95% [12].

Antimicrobial susceptibility testing (AST)

Initial AST was performed using the modified Kirby-Bauer disk diffusion method in accordance with the Clinical and Laboratory Standards Institute (CLSI) guidelines, 2023 edition (https://clsi.org/). Bacterial suspensions were standardized to a 0.5 McFarland turbidity standard before inoculation onto Mueller-Hinton agar plates. The following antibiotic discs were applied: amoxicillin-clavulanate (30 µg), piperacillin-tazobactam (100/10 µg), ceftriaxone (30 µg), cefepime (30 µg), imipenem (10 µg), meropenem (10 µg), ciprofloxacin (5 µg), levofloxacin (5 µg), amikacin (30 µg), gentamicin (10 µg), trimethoprim-sulfamethoxazole (1.25/23.75 µg), chloramphenicol (30 µg), fosfomycin (200 µg), tigecycline (15 µg), and CZA (30/20 µg). Following 24 hours of aerobic incubation at 37 °C, zones of inhibition were measured and interpreted as susceptible, intermediate, or resistant, based on CLSI interpretive criteria [13].

Minimum inhibitory concentration (MIC) of CZA

MICs of CZA were determined using the automated VITEK® 2 Compact system (bioMérieux SA) with GN AST-419 cards, following the manufacturer's instructions. Standardized bacterial suspensions equivalent to 0.5 McFarland were prepared and loaded into the system. MICs were automatically interpreted based on kinetic growth curves and classified according to CLSI M100 guidelines, 34th edition (2024). Interpretations were expressed as susceptible (≤ 8 μg/mL), intermediate (8-16 μg/mL), or resistant (≥ 328 μg/mL) [14].

Genomic DNA extraction

Genomic DNA from the CZA-resistant *K. pneumoniae* was extracted by inoculating a single, well-isolated colony of *K. pneumoniae*, obtained from selective culture media, into 5 mL of Luria-Bertani (LB) broth, followed by incubation at 37 °C for 18-24 hours under aerobic conditions to achieve optimal biomass. The bacterial cells were subsequently harvested via high-speed centrifugation at 16,000 × g for five minutes. The supernatant was discarded, and the resulting pellet was resuspended in 50 μL of nuclease-free water. Cell lysis was induced by thermal disruption through incubation at 95 °C for seven minutes. To remove insoluble cellular debris, the lysate was centrifuged at 8,000 × g for five minutes. The resulting supernatant, containing crude genomic DNA, was carefully collected and employed as the template for polymerase chain reaction (PCR) and other downstream molecular analyses [15].

PCR amplification for *bla_KPC_* and *bla_CMY_* AmpC genes

PCR was performed for the detection of KPC-2 and CMY AmpC genes using specific primers [15]. The primer sequences and expected amplicon sizes for both genes are provided in Table 1.

**Table 1:** Primer sequences and expected amplicon size for blaKPC-2 and blaCMY AmpC genes

Primers	Primer Sequence (5’-3’)	Amplicon size
bla_KPC-F_	CGTCTAGTTCTGCTGTCTTG	798 bp
bla_KPC-R_	CTTGTCATCCTTGTTAGGCG	798 bp
bla_CMY-F_	AACACACTGATTGCGTCTGAC	1226 bp
bla_CMY-R_	CTGGGC CTCATCGTCAGTTA	1226 bp

Conventional PCR was carried out in a final reaction volume of 25 µL. The reaction mixture comprised 2 µL of genomic DNA template, 12.5 µL of PCR Master Mix (containing 10× reaction buffer, 2.5 mM MgCl₂, 200 µM of each deoxynucleotide triphosphate (dNTP), and 1 unit of Taq DNA polymerase), 1 µL each of forward and reverse primers (10 pmol), and 8.5 µL of nuclease-free water.

For the amplification of the *bla_KPC-2_* gene, the thermal cycling conditions were as follows: initial denaturation at 94 °C for five minutes, followed by 30 amplification cycles consisting of denaturation at 94 °C for 30 seconds, primer annealing at 52 °C for 40 seconds, and extension at 72 °C for 50 seconds. A final extension was performed at 72 °C for seven minutes [16].

For the detection of the *bla_CMY_* AmpC gene, PCR conditions included an initial denaturation at 95 °C for five minutes, followed by 36 cycles of denaturation at 94 °C for 45 seconds, annealing at 53 °C for one minute, and extension at 72 °C for one minute. The final extension was carried out at 72 °C for seven minutes.

Agarose gel electrophoresis

Following amplification, PCR products were resolved by electrophoresis on a 1.5% (w/v) agarose gel prepared in 1× Tris-acetate-EDTA (TAE) buffer. The gel was stained with ethidium bromide (EtBr) and visualized under ultraviolet (UV) transillumination to confirm the presence and size of the amplified DNA fragments.

Sequence analysis

A purified amplicon of the *bla_CMY_* gene, amplified from a CZA-resistant *K. pneumoniae* isolate, was subjected to bidirectional Sanger sequencing using the original primer set. Sequencing was performed by a certified commercial provider (e.g., Macrogen Inc., South Korea). Chromatographic data were quality-checked using Chromas Lite v2.6.6 (Technelysium Pty. Ltd, Queensland, Australia), and a consensus sequence was assembled in BioEdit (Informer Technologies, Inc., Los Angeles, California, United States). Nucleotide identity was confirmed via BLASTn analysis against the NCBI GenBank database (https://www.ncbi.nlm.nih.gov/genbank/). Subsequent multiple sequence alignment using ClustalW in MEGA X (https://www.megasoftware.net/) demonstrated 99.8% identity with the reference *bla_CMY-2_* allele. No non-synonymous substitutions, indels, or frameshift mutations were observed, and the coding sequence remained intact. The verified sequence was deposited in GenBank under submission number "2983196".

Statistical analysis

All collected data were compiled using Microsoft Excel (Microsoft Corporation, Redmond, Washington, United States) and analyzed with IBM SPSS Statistics for Windows, version 20 (IBM Corp., Armonk, New York, United States). Descriptive statistics, including frequencies and percentages, were calculated to evaluate the resistance profile, as well as the detection rates of *bla_KPC-2_* and *bla_CMY_* AmpC genes identified by PCR.

To assess the association between the presence of β-lactamase genes and phenotypic resistance, the chi-square test was applied. Where expected cell counts were below five, Fisher’s exact test was used as an alternative. A p-value < 0.05 was considered statistically significant for all analyses, indicating a meaningful correlation between genotypic findings and antimicrobial resistance profiles.

## Results

Distribution of specimens

**Figure 1:**
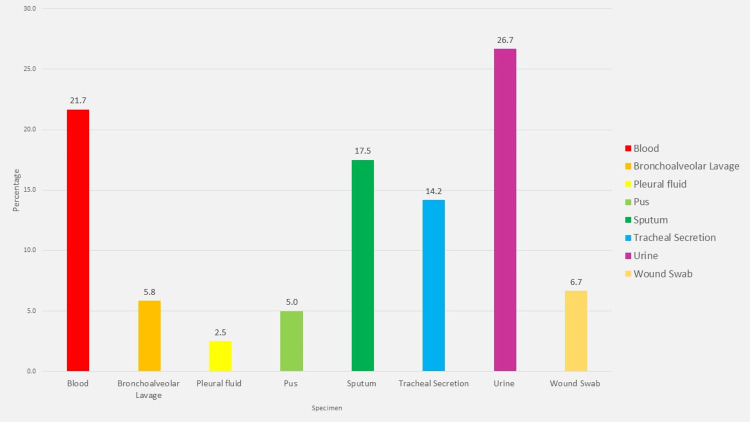
Specimen wise distribution of Klebsiella pneumoniae Specimen-wise distribution of *Klebsiella pneumonia*e isolates (n = 120). Urine was the predominant source, followed by blood, sputum, tracheal secretions, Pleural, and other body fluids

Among the 120 *K. pneumoniae* isolates recovered during the study period, as shown in Figure 1, the majority originated from urine specimens, comprising 26.7% (n = 32) of the total. Blood cultures represented the second most prevalent source at 21.7% (n = 26). Respiratory tract specimens also yielded a substantial number of isolates, with sputum accounting for 17.5% (n = 21), tracheal secretions 14.2% (n = 17), and bronchoalveolar lavages 5.8% (n = 7). Isolates from wound swabs and pus samples contributed 6.7% (n = 8) and 5.0% (n = 6), respectively. Additionally, 8.3% (n = 10) of isolates were derived from sterile body fluids, including pleural fluid, which accounted for 2.5% (n = 3). 

**Figure 2:**
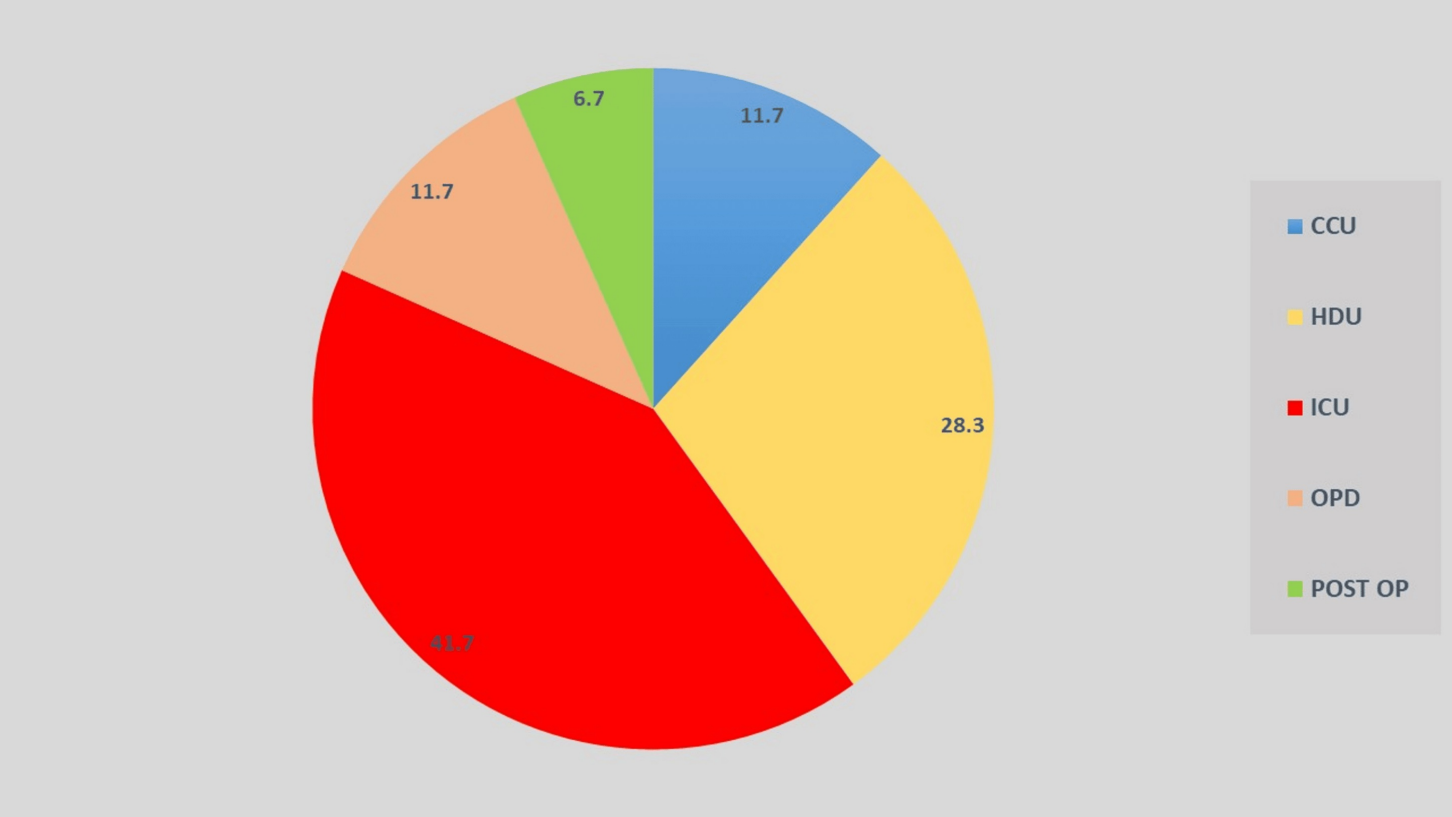
Department-wise distribution of clinical isolates ICU: intensive care unit; HCU: high dependency unit; CCU: critical care unit; OPD: outpatient department; post op: postoperative ward

Departmental analysis (Figure 2) showed that the highest proportion of isolates originated from patients admitted to the intensive care unit (ICU), comprising 41.7% (n = 50) of the total isolates. This was followed by the high dependency unit (HDU) with 28.3% (n = 34) and the coronary care unit (CCU) with 11.7% (n = 14). Isolates recovered from the Outpatient Department (OPD) also accounted for 11.7% (n = 14), while those from the Postoperative Ward constituted 6.7% (n = 8). The predominance of isolates from CCUs highlights the nosocomial potential of *K. pneumoniae*, particularly in vulnerable and immunocompromised patient populations, reaffirming its role as a key pathogen in healthcare-associated infections.

Antimicrobial susceptibility patterns

**Figure 3:**
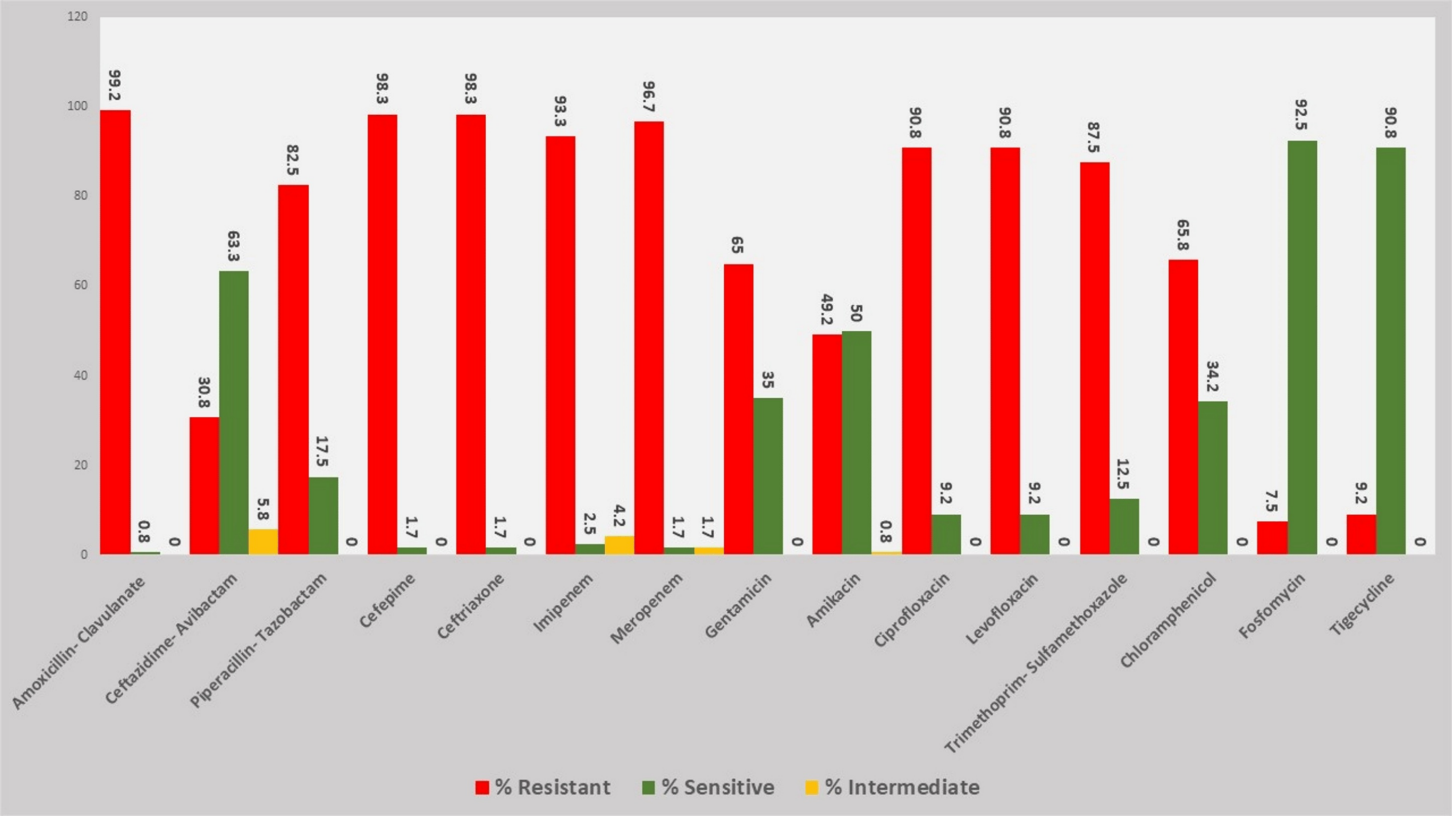
Antimicrobial resistance profile of Klebsiella pneumoniae isolates Antimicrobial resistance profile of *Klebsiella pneumoniae* isolates (n = 120) based on disc diffusion testing. The bar chart illustrates the percentage distribution of resistant (red), intermediate (yellow), and susceptible (green) isolates against the tested antibiotics. Resistance was most prevalent for β-lactams and third and fourth-generation cephalosporins, carbapenem, intermediate sensitivity against amikacin, gentamicin, and chloramphenicol, with relatively higher susceptibility observed for tigecycline and fosfomycin and ceftazidime-avibactam show 30.8% resistance

Among the *K. pneumoniae* isolates, resistance was observed across multiple antibiotic classes, including β-lactam, cephalosporins, carbapenems, and fluoroquinolones (Table 2 and Figure 3). The highest resistance rate was observed against amoxicillin-clavulanate (20/10 µg), with 119 out of 120 isolates (99.2%) exhibiting resistance. Cephalosporin resistance was also significant, with ceftriaxone (30 µg) and cefepime (30 µg) showing resistance in 118 isolates (98.3%) each. Among carbapenems, resistance to meropenem (10 µg) and imipenem (30 µg) was noted in 116 isolates (96.7%) and 112 isolates (93.3%), respectively. Fluoroquinolones demonstrated substantial resistance as well, with ciprofloxacin (5 µg) and levofloxacin (5 µg) exhibiting non-susceptibility in 109 isolates (90.8%). Piperacillin-tazobactam (100/10 µg) resistance was detected in 99 isolates (82.5%). Moderate resistance rates were observed for gentamicin (10 µg) at 65.0% (78/120), chloramphenicol (30 µg) at 65.8% (79/120), amikacin (30 µg) at 49.2% (60/120), and trimethoprim-sulfamethoxazole (1.25/23.75 µg) at 87.5% (105/120). Comparatively lower resistance rates were observed for CZA (30/20 µg), with 37 isolates (30.8%) classified as resistant. Resistance to tigecycline (15 µg) and fosfomycin (200 µg) was detected in only 11 (9.2%) and nine (7.5%) isolates, respectively, suggesting retained efficacy of these agents against a substantial proportion of isolates. Intermediate susceptibility was rarely observed and was limited to a few agents: CZA (5.8%), imipenem (4.2%), meropenem (1.7%), and amikacin (0.8%). These findings underscore the predominance of full resistance phenotypes among the tested *K. pneumoniae* isolates.

**Table 2:** Antimicrobial susceptibility profile of Klebsiella pneumoniae Antimicrobial susceptibility profile of *Klebsiella pneumoniae* isolates (n = 120). High resistance rates were observed against β-lactams, cephalosporins, and carbapenems, while aminoglycosides, tigecycline and fosfomycin retained activity against most isolates.

Antibiotic (Disc Potency)	Resistant, n (%)	Intermediate, n (%)	Susceptible, n (%)
Amoxicillin-Clavulanate (20/10 µg)	119 (99.2%)	1 (0.8%)	0 (0.0%)
Ceftriaxone (30 µg)	118 (98.3%)	2 (1.7%)	0 (0.0%)
Cefepime (30 µg)	118 (98.3%)	2 (1.7%)	0 (0.0%)
Meropenem (10 µg)	116 (96.7%)	2 (1.7%)	2 (1.6%)
Imipenem (30 µg)	112 (93.3%)	5 (4.2%)	3 (2.5%)
Ciprofloxacin (5 µg)	109 (90.8%)	6 (5.0%)	5 (4.2%)
Levofloxacin (5 µg)	109 (90.8%)	6 (5.0%)	5 (4.2%)
Piperacillin-Tazobactam (100/10 µg)	99 (82.5%)	10 (8.3%)	11 (9.2%)
Trimethoprim-Sulfamethoxazole (1.25/23.75 µg)	105 (87.5%)	7 (5.8%)	8 (6.7%)
Gentamicin (10 µg)	78 (65.0%)	8 (6.7%)	34 (28.3%)
Chloramphenicol (30 µg)	79 (65.8%)	6 (5.0%)	35 (29.2%)
Amikacin (30 µg)	60 (49.2%)	1 (0.8%)	59 (49.2%)
Ceftazidime-Avibactam (30/20 µg)	37 (30.8%)	7 (5.8%)	76 (63.4%)
Tigecycline (15 µg)	11 (9.2%)	4 (3.3%)	105 (87.5%)
Fosfomycin (200 µg)	9 (7.5%)	3 (2.5%)	108 (90.0%)

MICs of CZA

**Figure 4:**
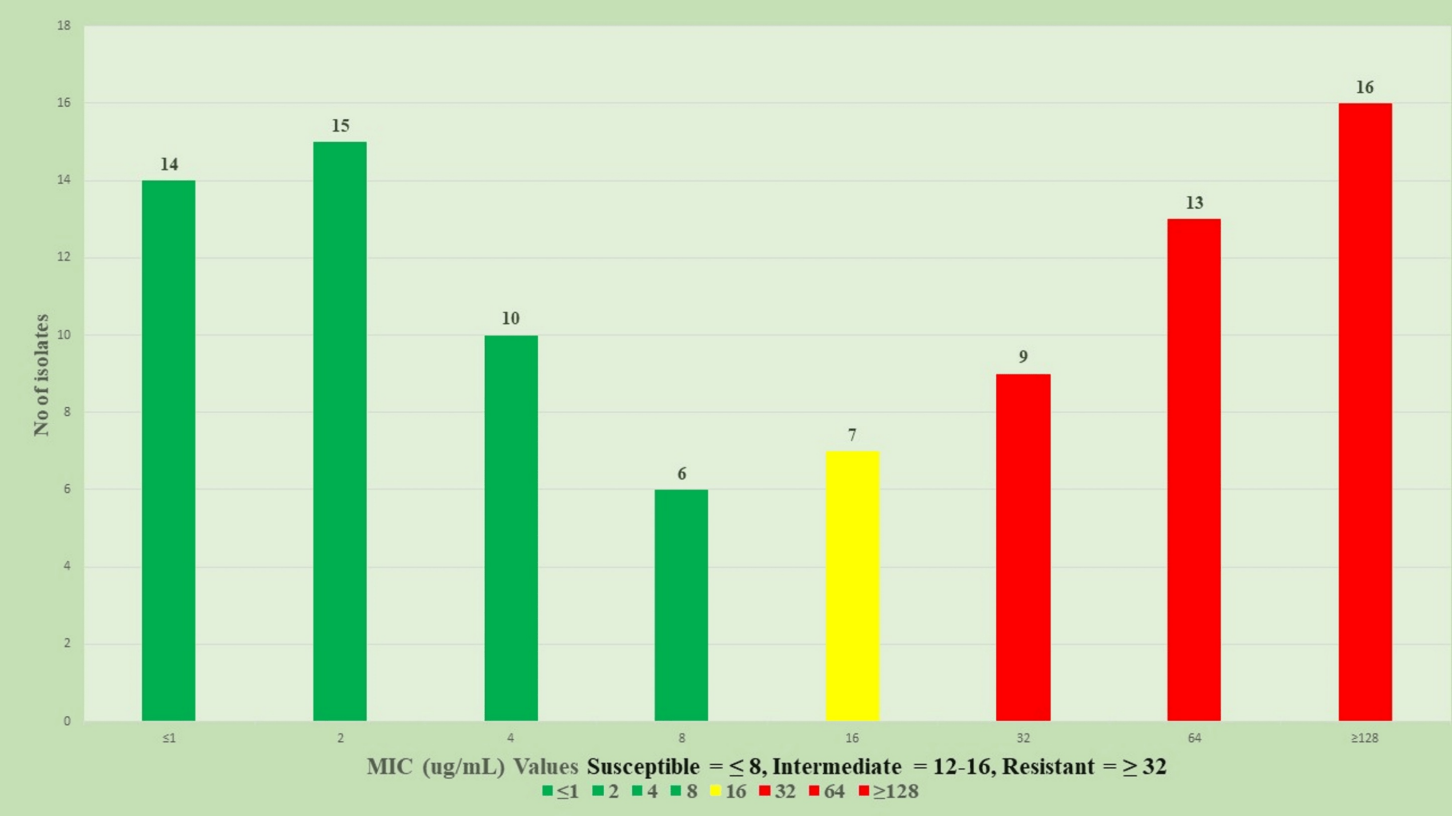
MIC distribution of ceftazidime-avibactam among Klebsiella pneumoniae isolates Minium Inhibitory concentration (MIC) distribution of ceftazidime-avibactam among *Klebsiella pneumoniae* isolates (n = 120). Isolates were classified as susceptible (≤8 µg/mL), intermediate (12–16 µg/mL), and resistant (≥32 µg/mL) based on CLSI 2023 breakpoints. A substantial proportion exhibited high-level resistance, with MICs ≥128 µg/mL.

The MIC values of CZA were determined for all 120 *K. pneumoniae* isolates using the VITEK 2 automated system and interpreted according to CLSI 2023 breakpoints. Of the total isolates, 76 (63.3%) were classified as susceptible, seven (5.8%) as intermediate, and 37 (30.8%) as resistant, as shown in Figure 4.

The susceptible group exhibited low MICs, with a mean MIC of 3.42 ± 2.38 µg/mL, reflecting a favorable in vitro response to CZA. The intermediate isolates displayed uniform MIC values at the CLSI-defined intermediate threshold, resulting in a mean MIC of 16.00 ± 0.00 µg/mL. In contrast, the resistant group demonstrated markedly elevated MICs, with a mean MIC of 91.46 ± 24.36 µg/mL, including multiple isolates exhibiting values at or above ≥128 µg/mL, suggestive of high-level resistance. These findings underscore the heterogeneous susceptibility pattern of *K. pneumoniae* to CZA and highlight a considerable proportion of isolates with clinically significant resistance, necessitating further genomic and therapeutic scrutiny.

Frequency of *bla_CMY_* AmpC and *bla_KPC-2_* genes

**Figure 5:**
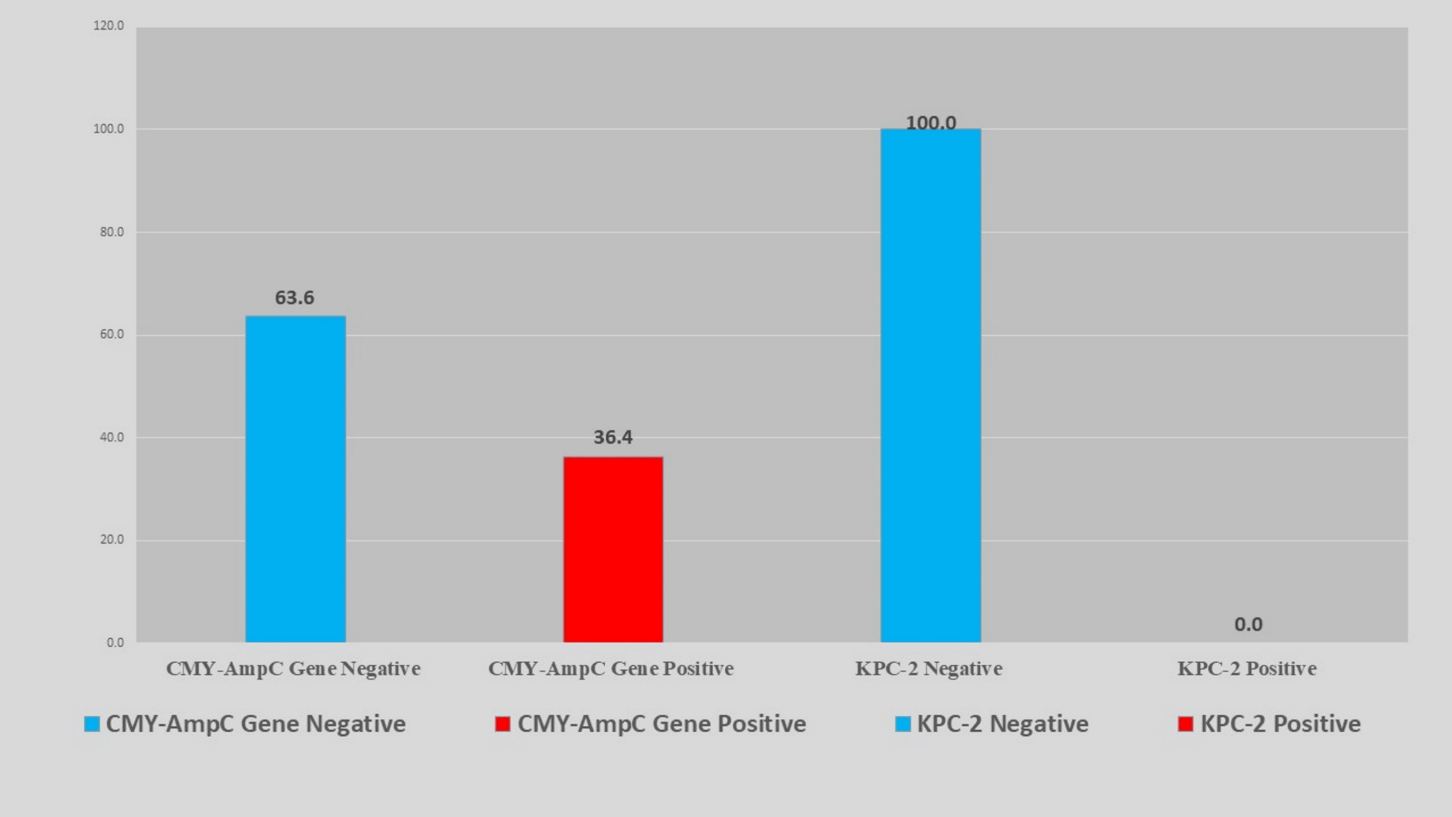
Distribution of blaCMY and blaKPC-2 genes detected by PCR in non-susceptible Klebsiella pneumoniae isolates. A substantial portion carried *bla_CMY_*, whereas *bla_KPC-2_* was absent. PCR: polymerase chain reaction

Figure 5 show that the *bla_CMY_* gene was detected in 16 isolates (36.4%), whereas 28 isolates (63.6%) were negative for this gene. Notably, none of the isolates (0%) tested positive for *bla_KPC-2_*, indicating the complete absence of KPC in this cohort.

Co-Existence Patterns

No isolate demonstrated co-carriage of both resistance genes. The *bla_CMY_* gene was detected in isolation, and *bla_KPC-2_* was not detected in all tested samples. As a result, no co-existence of *bla_CMY_* and *bla_KPC-2_* was observed among the study isolates

Association with Resistance Profiles

All *bla_CMY_* positive isolates (n = 16) exhibited high MIC values for CZA and were classified within the resistant category. In contrast, none of the isolates displaying intermediate (n = 7) or sensitive (n = 76) MIC profiles were positive for either resistance gene.

Statistical analysis revealed a significant association between the presence of the *bla_CMY_* gene and CZA-resistance (p < 0.05). This suggests that *bla_CMY_* positivity was exclusively distributed among phenotypically resistant isolates. Conversely, no statistical correlation could be established for *bla_KPC-2_* as it was not detected in any of the 44 non-susceptible isolates tested.

Sequencing confirmation

To validate the PCR findings, one *bla_CMY_*-positive isolate was subjected to gene sequencing. The amplicon showed 99.3% nucleotide identity with the *bla_CMY-2_* gene deposited in the NCBI GenBank database, thereby confirming the specificity and accuracy of the molecular detection protocol.

## Discussion

The present study reveals a critically high prevalence of MDR *K. pneumoniae* among clinical isolates, especially in critical areas, with resistance to meropenem and imipenem observed in 96.7% and 93.3% of cases, respectively. Furthermore, over 98% of isolates exhibited resistance to third and fourth-generation cephalosporins. These findings are consistent with resistance patterns typically reported in resource-limited healthcare systems, where antimicrobial stewardship programs and infection control measures may be inadequately implemented [17].

In line with regional trends, a multicenter study from Iran in 2020 reported a pooled prevalence of carbapenem-resistant *K. pneumoniae* of approximately 24%, indicating a significant rise in resistance rates compared to previous decades and underscoring the growing clinical threat of carbapenemase-producing Enterobacterales [18]. Between 2022 and 2024, antimicrobial resistance surveillance in South India revealed that approximately one-quarter (24%) of gram-negative bacilli were resistant to carbapenems, with *K. pneumoniae* comprising 39% of the carbapenem-resistant isolates [11]. Although regional reports describe considerable resistance burdens, the near-complete carbapenem resistance observed in our study underscores a more alarming scenario for treating critically ill patients. In contrast, clinical surveillance programs in Western Europe and North America report substantially lower frequencies of resistance to advanced β-lactam/β-lactamase inhibitor combinations [13]. Specifically, CZA resistance has been documented at rates consistently under 10% in multiple systematic evaluations conducted in the United States, Italy, and Greece [13,19].

In this study, a CZA resistance rate of 30.8% was observed among *K. pneumoniae* isolates, reflecting trends reported across parts of Asia. Similarly, a recent global meta-analysis revealed that 29.4% of carbapenem-resistant *K. pneumoniae *isolates exhibited CZA resistance, largely driven by CMY-type AmpC β-lactamases [15]. According to Wang et al., resistance among ICU-derived Klebsiella pneumoniae isolates reached 32.1%, primarily driven by plasmid-borne AmpC β-lactamases and impaired outer membrane permeability resulting from porin mutations [20]. Similar trends have been observed in European studies, where AmpC overexpression and structural alterations in porins were identified as key contributors to elevated CZA resistance levels [21].

In our cross-sectional study, *K. pneumoniae* isolates exhibited mean MICs for CZA of 3.2 µg/mL for susceptible isolates, 16.3 µg/mL for intermediate, and 70.8 µg/mL for resistant isolates, based on automated MIC testing. These findings align with the 2024 European Committee on Antimicrobial Susceptibility Testing (EUCAST) clinical breakpoints, where an MIC of ≥32 µg/mL is categorized as resistant, correlating with a higher risk of poor clinical outcomes [22]. Similarly, Liu et al. reported that more than 80% of CZA-resistant *K. pneumoniae* isolates recovered from critically ill patients exhibited MIC values of ≥64 µg/mL, underscoring the strong correlation between elevated MICs and therapeutic failure in intensive care settings [23]. These observations further support the clinical relevance of MIC determination, not only for laboratory categorization but also for optimizing treatment decisions, highlighting the necessity of susceptibility-guided therapy before the empirical use of CZA in high-risk patient populations.

Among the CZA-resistant *K. pneumoniae* isolates analyzed in this study, 36.4% were found to harbor the *bla_CMY_* gene, while none tested positive for the *bla_KPC-2_* gene. This molecular profile is clinically significant, as recent studies have increasingly highlighted the role of AmpC β-lactamases, particularly CMY enzymes, as emerging determinants of resistance to CZA, in contrast to the historically predominant KPC-mediated mechanisms. The absence of *bla_KPC-2_* in our resistant isolates underscores a shifting resistance landscape, wherein plasmid-mediated AmpC production may be an under-recognized but critical contributor to therapeutic failure with novel β-lactam/β-lactamase inhibitor combinations [21]. In contrast to earlier findings from regions such as China and the United States, where CZA resistance has been primarily associated with KPC-type β-lactamases, often involving Ω-loop mutations (e.g., KPC-33, KPC-51, KPC-52, KPC-90) [7,16,24], our findings align more closely with emerging resistance trends observed in South Asian and Middle Eastern isolates, suggesting a regional shift in the molecular epidemiology of resistance mechanisms [10-12]. Nationwide studies from China have linked various *bla_KPC_* allelic variants to CZA resistance, often coupled with porin loss and gene amplification. Emerging surveillance data from South Asia indicate a surge in plasmid-mediated AmpC β-lactamases, notably CMY-2, among *K. pneumoniae* isolates, highlighting a shift towards non-KPC resistance profiles [10,12]. These CMY enzymes are commonly encoded on mobile genetic elements such as conjugative plasmids, promoting their rapid transfer and spread in clinical environments [25]. The dissemination of CMY β-lactamase-mediated resistance, often linked to conjugative plasmids, enhances horizontal gene transfer and accelerates spread among clinical pathogens [26]. Our data, alongside regional reports, reflect a paradigm shift in *K. pneumoniae* resistance patterns toward plasmid-borne AmpC β-lactamases, particularly CMY variants, posing a direct threat to conventional KPC-focused diagnostics and therapeutics. We recommend strengthening molecular diagnostic capacity and implementing sustained genomic surveillance to guide therapy and help preserve the effectiveness of novel β-lactam/β-lactamase inhibitor combinations.

Our study demonstrated a statistically significant association between CMY AmpC β-lactamase-positive and phenotypic resistance to CZA (p < 0.05). All *bla_CMY_* positive isolates (n = 16) exhibited MICs ≥ 32 µg/mL, consistent with high-level resistance, while none of the susceptible or intermediate isolates harbored either *bla_CMY_* or *bla_KPC‑2_*. This genotype-phenotype correlation aligns with structural and epidemiological observations of CMY-mediated CZA resistance in Enterobacterales [27,19].

CZA resistance in *K. pneumoniae* is predominantly associated with overexpression of AmpC β-lactamases, structural mutations within the Ω-loop of KPC enzymes (notably D179Y substitutions in variants like KPC-33 and KPC-44), outer membrane porin disruptions (OmpK35/36), and upregulation of efflux mechanisms [28,29]. Emerging evidence from China and adjacent regions demonstrates a rising prevalence of plasmid-mediated AmpC β-lactamases, particularly CMY-2, among *K. pneumoniae* isolates, reflecting an evolving shift from KPC-dominated to diverse non-KPC resistance mechanisms [15,16]. These CMY variants, frequently harbored by high-risk clones such as ST11, exhibit active site alterations that diminish avibactam’s inhibitory efficacy while preserving bacterial viability, often resulting in markedly elevated MIC values (up to 128 mg/L) [20].

In light of the substantial prevalence of *bla_CMY_*-mediated resistance to CZA observed in this study, its empirical use should be approached with caution, particularly in high-risk clinical settings. Alternative agents such as tigecycline and fosfomycin, which demonstrated resistance rates below 10% among our isolates, may offer viable therapeutic options; however, their pharmacokinetic limitations and restricted clinical indications necessitate judicious application within tailored antimicrobial regimens [30,31].

This study is limited by a single-center design, moderate sample size, and a narrow molecular scope, focusing only on *bla_CMY_* and *bla_KPC-2_*. Other potential resistance mechanisms, including *bla_NDM_*, OXA-48-like enzymes, extended-spectrum beta-lactamase (ESBLs), porin alterations, efflux pump activity, and genomic epidemiology, were not evaluated and may influence the broader resistance profile.

## Conclusions

In this study, plasmid-mediated CMY AmpC β-lactamase emerged as the main mechanism of CZA resistance in *K. pneumoniae*, while no *bla_KPC-2_* was detected. Our findings suggest a possible regional trend in antimicrobial resistance rather than a definitive regional shift, as the sample was derived from a single center, and the a need for routine AmpC gene screening to guide therapy and infection control. In such cases, alternatives such as cefiderocol, tigecycline, or fosfomycin may be considered. Continued genomic surveillance is essential to track resistance trends and support stewardship efforts.
